# *In-silico* identification of linear B-cell epitopes in specific proteins of *Bartonella bacilliformis* for the serological diagnosis of Carrion’s disease

**DOI:** 10.1371/journal.pntd.0011321

**Published:** 2023-05-25

**Authors:** Victor Jimenez-Vasquez, Karen Daphne Calvay-Sanchez, Yanina Zarate-Sulca, Giovanna Mendoza-Mujica

**Affiliations:** Laboratory of Vector-Borne Bacterial Diseases. National Institute of Health, Lima, Peru; Institut Pasteur, FRANCE

## Abstract

Carrion´s disease is caused by *Bartonella bacilliformis*, it is a Gram-negative pleomorphic bacterium. *B*. *bacilliformis* is transmitted by *Lutzomyia verrucarum* in endemic areas of the Peruvian Inter-Andean valleys. Additionally, the pathogenicity of *B*. *bacilliformis* involves an initial infection of erythrocytes and the further infection of endothelial cells, which mainly affects children and expectant women from extreme poverty rural areas. Therefore, the implementation of serological diagnostic methods and the development of candidate vaccines for the control of CD could be facilitated by the prediction of linear b-cell epitopes in specific proteins of *B*. *bacilliformis* by bioinformatics analysis. In this study, We used an *in-silico* analysis employing six web servers for the identification of epitopes in proteins of *B*. *bacilliformis*. The selection of *B*. *bacilliformis-*specific proteins and their analysis to identify epitopes allowed the selection of seven protein candidates that are expected to have high antigenic activity.

## Author summary

Carrion´s disease (CD) affects poor communities from the Andean region in South America, where lethal cases have been reported and associated with hemolytic anemia. Therefore, early-stage diagnosis is relevant to treat the early stage of infection, the diagnostic alternatives include serological tests or molecular assays. The serological assays are wildly used because of their low cost and easy application, hence the improvement of the sensitivity and specificity of the serological assays provides better accuracy for the diagnosis CD. In this study, we look for the prediction of specific proteins to *B*. *bacilliformis*, the causative agent of CD, and the identification of lineal B-epitopes by *in-silico* analysis. Using a six-web server, we identified linear epitopes in seven specific proteins which are expected to have antigenic activity.

## Introduction

Carrion’s disease (CD) is an endemic disease of the Andean countries such as Peru and Ecuador, and was formerly reported in Colombia [1,2]. CD represents one of the main challenges for public health due to poverty and poor sanitation in endemic localities, affecting children with chronic malnutrition. *Bartonella bacilliformis*, the etiological agent of CD, is a Gram-negative pleomorphic bacterium transmitted by sand flies of the *Lutzomyia* genus, especially *L*. *verrucarum* [2]. Climate change and variability in inter-Andean valley rainfall associated with the El Niño-Southern Oscillation (ENSO) contribute to the reproduction of the associated vector and the emergence of local CD outbreaks with a non-negligible number of cases [3].

CD clinical manifestations are diverse as the microorganism parasitizes human erythrocytes generating an acute phase, named Oroya fever, characterized by anemia and febrile illness [2], nevertheless, the nature of the initial symptoms may be confused with that of other infectious diseases, such as malaria, dengue or others. This is of special relevance because the absence or delay of adequate treatment may result in fatal outcomes, hence mortality rates among untreated or inadequately treated patients were described as up to 88% [2,4]. In Peru, the overall Oroya fever lethality ranges between 0.5 and 3%, with about 10% of severe cases attending reference hospitals having a fatal outcome [2]. After a period of several weeks following the acute phase, the patients display a non-life threatening eruptive phase termed verrucose phase [1,2]. The eruptive phase is characterized by skin eruptions and may be presented in the absence of a previous acute infection [1,2]. Although, patients with asymptomatic bacteremia have been identified, they are considered as potential reservoirs of *B*. *bacilliformis* and a potential source of infection for susceptible persons [3].

In relation to the diagnosis of CD, bacteriological cultures are accurate, but they are time-consuming because of the slowness in the colony formation by *B*. *bacilliformis* (about 4–15 days) which limits its usefulness for diagnostic purposes. On the other hand, blood smear detection is widely used due to its accuracy and low cost, but it has a low sensitivity that can generate false negatives that affect the confirmation of the diagnosis [5,6]. Molecular methods based on the amplification of specific gene regions such as *gltA*, *ribC* and *ialB* have been proposed, however, the implementation of these techniques in rural areas is challenging due to the lack of equipment, and some of these genes are not specific for *B*. *bacilliformis*, causing cross reactivity with other pathogens [7].

Furthermore, the available serological technique used for the diagnosis of CD employs soluble protein lysate, it displays limited specificity, and potential cross-reactivity with other *Bartonella*ceae or other microorganisms, similar to what has been reported for different immunological approaches for the detection of *Bartonella quintana* and *Bartonella henselae* infections [8,9]. Regarding rapid diagnostic tools that can be used in endemic areas, different antigenic candidates have been proposed but none has been introduced in clinical practice [10,11]. Data about the immune response to *B*. *bacilliformis* is scarce, antibody immunity build-up for the development of partial immunity [10,12]. Thus, immunoglobulin M (IgM) is considered as a biomarker of the acute phase and immunoglobulin G (IgG) as a marker of previous exposure [10,12].

Identification of the antigenic-determinant is crucial for designing immune treatment such as a vaccine against infectious diseases, or to avoid cross-reactivity of antibodies used in diagnostic methods [13]. Immunoinformatics addresses the molecular interactions of potential binding sites by computational methods [14] and is the best approach to recognizing epitopes. Immunoinformatics is inexpensive, accurate and not time-consuming, allowing the design and synthesis of a molecule that can be used as an antigen [13]. Hence, the present study aimed to identify linear B-cell epitopes in specific proteins of *B*. *bacilliformis*.

## Materials and methods

### Data preparation

Proteins highly enriched in linear B-cell of *B*. *bacilliformis* based on the complete genome of the strain KC584 (GenBank code CP045671.1) were identified [15]. The selected genome contains 1’411´655 base pairs, displays a 500.0 X genome coverage, and was obtained by PacBio Sequel and Illumina MiSeq [15]. Functional genes (CDSs) were downloaded in FASTA format and all headers were edited in order to retain the name, orientation and position in the genome.

The *B*. *bacilliformis* strain KC584 was analyzed using Database of Essential Genes (http://origin.tubic.org/deg/public/index.php) [16]. The essential genes indispensable for the survival of an organism were identified, nucleotide sequences were translated into amino acid sequences with the VirtualRibsome 2.0 bioinformatic tool [17] (https://services.healthtech.dtu.dk/service.php?VirtualRibosome-2.0), sequences were downloaded in FASTA format.

### Selection of non-homologous proteins

To obtain only *B*. *bacilliformis*-exclusive-proteins, we used the BLAST+ tool to identify non-homologous proteins, comparing proteins encoded by the essential genes *of B*. *bacilliformis* to the proteome of *Homo sapiens*, *Mus musculus*, febrile illness-associated bacterial species and other *Bartonella* species. First at all, We identified non homologous proteins shared with *Homo sapiens* (GenBank code GRCh38p.13) and *Mus musculus* (GenBank code GRCm39), to avoid cross-reactivity when human samples and animal model samples are used, respectively. The selected parameters were Bit-score < 100, % identity < 35, % coverage < 35.

Further analyses were performed to apply second selection criteria, the identification of non-homologous proteins shared with others bartonellae was performed considering Bit-score < 100, identity < 95%, coverage < 95%. Finally, febrile illness-associated bacterial species belonging to genera *Anaplasma*, *Coxiella*, *Brucella*, *Ehrlichia*, *Leptospira*, *Neorickettsia*, *Orientia* and *Rickettsia* were faced with *B*. *bacilliformis* to identify non-homologous proteins, using Bit-score < 100, identity < 95% and < coverage < 95%. Thresholds were subjected to our specific results.

Finally, identity percentages were considered as a parameter for the selection of *B*. *bacilliformis* specific proteins, considering proteins with less than 80% identity to other bartonellae and 70% identity to fever-causing bacteria.

### *In-silico* characterization of non-homologous proteins

The non-homologous proteins were characterized to determine presence and location of signal peptide, cellular localization, and functionality, by using several bioinformatic tools to improve the predictive analysis. Biological function and gene ontology was predicted using EggNOG 5.0 (http://eggnog5.embl.de/#/app/home) [18]. Subcellular localizations were inferred with Cello [19] (http://cello.life.nctu.edu.tw/cello2go/) and Psort-B 3.0 [20] (https://www.psort.org/psortb/). Signal peptides were detected with PrediSi [21] (http://www.predisi.de/), SignalP 5.0 [22] (http://www.cbs.dtu.dk/services/SignalP/) and Phobius [23, 24] (https://phobius.sbc.su.se/) predictors.

### Identification of linear B-cell epitopes in non-homologous proteins

The location of linear epitopes was predicted, and six web servers were used to improve the accuracy of the prediction: SVMTrip (http://sysbio.unl.edu/SVMTriP/prediction.php) [25], LBTope (http://webs.iiitd.edu.in/raghava/lbtope/protein.php) [26], BepiPRED2 (http://www.cbs.dtu.dk/services/BepiPred/) [27], BCePRED (http://crdd.osdd.net/raghava/bcepred), [28], BCPREDS (http://ailab-projects1.ist.psu.edu:8080/bcpred/predict.html) [29], and ABCPred (http://crdd.osdd.net/raghava/abcpred/) [30]. Epitope scores obtained by every predictor per amino acid or peptide were normalized (scaled between 0 and 100) and epiptopic profiles were generated with R library ggplot2.

### *In-silico* characterization of potential protein candidates

The proteins highly enriched in linear B-cell epitopes were characterized, 3D structures were predicted with the Phyre2 online program [31] (http://www.sbg.bio.ic.ac.uk/phyre2), the 3D models were verified in Chimera [32] (www.cgl.ucsf.edu/chimera/). 

## Results

**Fig 1:**
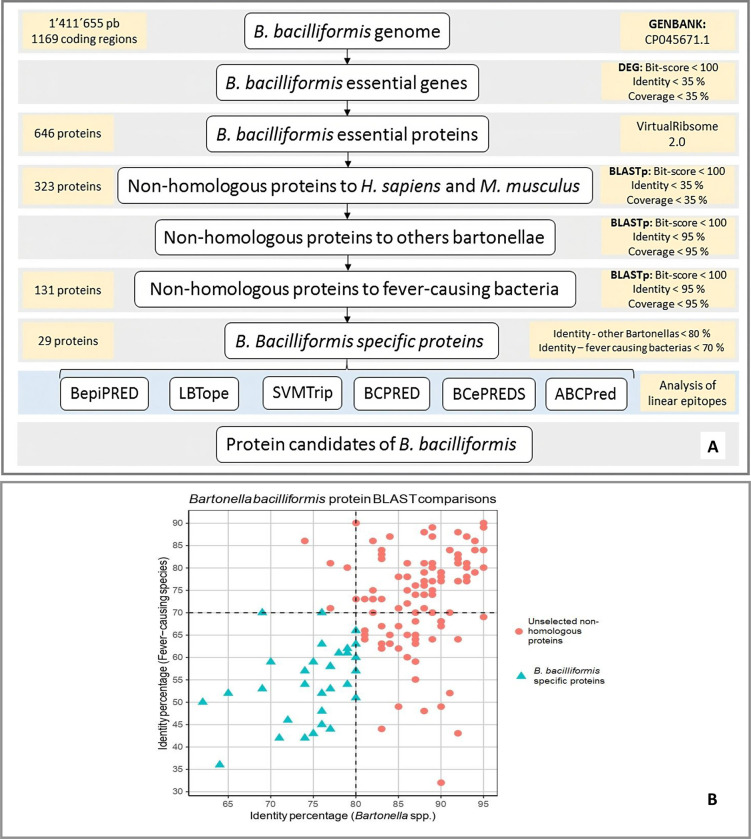
Workflow of *in-silico* analysis for selection of specific protein candidates. A) Screening workflow for selection of non-homologous proteins; B) Percentage identity of the 131 non-homologous proteins.

This study aimed to identify linear B-cell epitopes in highly specific proteins of *B*. *bacilliformis* for further use in serological diagnosis. The identification of essential genes allows for obtaining 646 proteins which were used for the screening of non-homologous proteins. Then, 323 proteins were obtained by comparing *B*. *bacilliformis* essential proteins with the proteome of *Homo sapiens* and *Mus musculus*, and 131 proteins were obtained and identified as non-homologous to febrile illness-associated bacterial species and other *Bartonella* species, see Fig 1.

Percentage identity was considered as a criterion for selection of *B*. *bacilliformis-*specific proteins, hence, 29 proteins were obtained and used to identify linear B-cell epitopes, see Table 1. The specific proteins include 13 cytoplasmic proteins, 12 cytoplasmic/membrane proteins, 2 outer membrane proteins, and 2 unknown-localization proteins, whilst the functionality of proteins involves more metabolic activities than structural conformation.

**Table 1:** *B*. *bacilliformis-*specific proteins obtained by *in-silico* analysis.

N°	Name	Accession number	Orientation	Coding Secuence		Protein length (amino acid)	Cellular localization, score*	Functionality
				Start	End			
**1**	Prot 30	QFZ89870.1	Pos	35684	38437	917	Cytoplasmic/Membrane, 10	Domain related to MnhB subunit of Na+/H+ antiporter
**2**	Prot 42	QFZ89881.1	Neg	47761	48849	362	Cytoplasmic/Membrane, 10	Beta-lactamase enzyme family
**3**	Prot 81	QFZ89919.1	Neg	92691	93659	322	Cytoplasmic, 9.97	Phage integrase, N-terminal SAM-like domain
**4**	Prot 91	QFZ89928.1	Pos	102638	105058	806	Cytoplasmic/Membrane,10	Ftsk_gamma
**5**	Prot 113	QFZ89950.1	Pos	131573	132574	333	Cytoplasmic/Membrane, 9.82	HlyD membrane-fusion protein of T1SS
**6**	Prot 116	QFZ90903.1	Pos	135752	136468	238	Cytoplasmic/Membrane, 10	Peptidoglycan polymerase that catalyzes glycan chain elongation from lipid-linked precursors
**7**	Prot 125	QFZ89960.1	Pos	144924	146753	609	Cytoplasmic, 9.97	ABC transporter C-terminal domain
**8**	Prot 137	QFZ89970.1	Neg	159365	160072	235	Cytoplasmic/Membrane, 10	MotA/TolQ/ExbB proton channel family
**9**	Prot 142	QFZ89975.1	Neg	163935	164453	172	Cytoplasmic, 8.96	Protein of unknown function (DUF1465)
**10**	Prot 213	QFZ90031.1	Neg	248142	250082	646	Cytoplasmic, 8.96	Peptidase family M23
**11**	Prot 232	QFZ90046.1	Pos	270825	272324	499	Cytoplasmic, 8.96	Unknown
**12**	Prot 236	QFZ90050.1	Pos	276314	278923	869	Cytoplasmic/Membrane, 9.99	Ftsk_gamma
**13**	Prot 288	QFZ90920.1	Neg	342093	342818	241	Cytoplasmic/Membrane, 9.82	ATPases associated with a variety of cellular activities
**14**	Prot 447	QFZ90250.1	Pos	524686	525879	397	Outer Membrane, 8.86	Lysin motif
**15**	Prot 492	QFZ90291.1	Neg	584045	585391	448	Cytoplasmic/Membrane, 9.82	Sporulation related domain
**16**	Prot 504	QFZ90301.1	Pos	596663	599059	798	Outer Membrane, 10	Part of the outer membrane protein assembly complex, which is involved in assembly and insertion of beta-barrel proteins into the outer membrane
**17**	Prot 612	QFZ90402.1	Pos	710016	710549	177	Cytoplasmic, 9.26	Single-strand binding protein family
**18**	Prot 679	QFZ90459.1	Pos	799102	800373	423	Cytoplasmic, 8.96	Uncharacterized protein family (UPF0051)
**19**	Prot 687	QFZ90466.1	Neg	808052	809095	347	Cytoplasmic, 9.97	DNA polymerase III, delta subunit
**20**	Prot 689	QFZ90468.1	Neg	810007	810852	281	Unknown,2	Lytic transglycosylase with a strong preference for naked glycan strands that lack stem peptides
**21**	Prot 733	QFZ90508.1	Pos	858296	859474	392	Cytoplasmic, 9.97	Exonuclease involved in the 3’ processing of various precursor tRNAs. Initiates hydrolysis at the 3’-terminus of an RNA molecule and releases 5’-mononucleotides
**22**	Prot 797	QFZ90569.1	Neg	949112	950530	472	Cytoplasmic, 9.97	Involved in cell wall formation. Catalyzes the final step in the synthesis of UDP-N-acetylmuramoyl-pentapeptide, the precursor of murein
**23**	Prot 810	QFZ90949.1	Neg	966711	968648	645	Cytoplasmic, 9.97	RNA polymerase that catalyzes the synthesis of short RNA molecules used as primers for DNA polymerase during DNA replication
**24**	Prot 1032	QFZ90774.1	Neg	1239066	1240526	486	Cytoplasmic/Membrane, 10	Probably responsible for the translocation of the substrate across the membrane
**25**	Prot 1041	QFZ90780.1	Pos	1248276	1250180	634	Cytoplasmic, 9.97	DNA polymerase III is a complex, multichain enzyme responsible for most of the replicative synthesis in bacteria. This DNA polymerase also exhibits 3’ to 5’ exonuclease activity
**26**	Prot 1078	QFZ90815.1	Neg	1287754	1288230	158	Cytoplasmic, 9.26	Catalyzes a trans-dehydration via an enolate intermediate
**27**	Prot 1084	QFZ90820.1	Pos	1296454	1297080	208	Cytoplasmic/Membrane, 10	Its exact role is uncertain. Responsible for energy coupling to the transport system
**28**	Prot 1088	QFZ90824.1	Pos	1298779	1299405	208	Unknown, 6.53	Redoxin
**29**	Prot 1149	QFZ90880.1	Pos	1385032	1387506	824	Cytoplasmic/Membrane, 9.9	PhoQ Sensor

*Score provided by Psort-B 3.0

**Fig 2:**
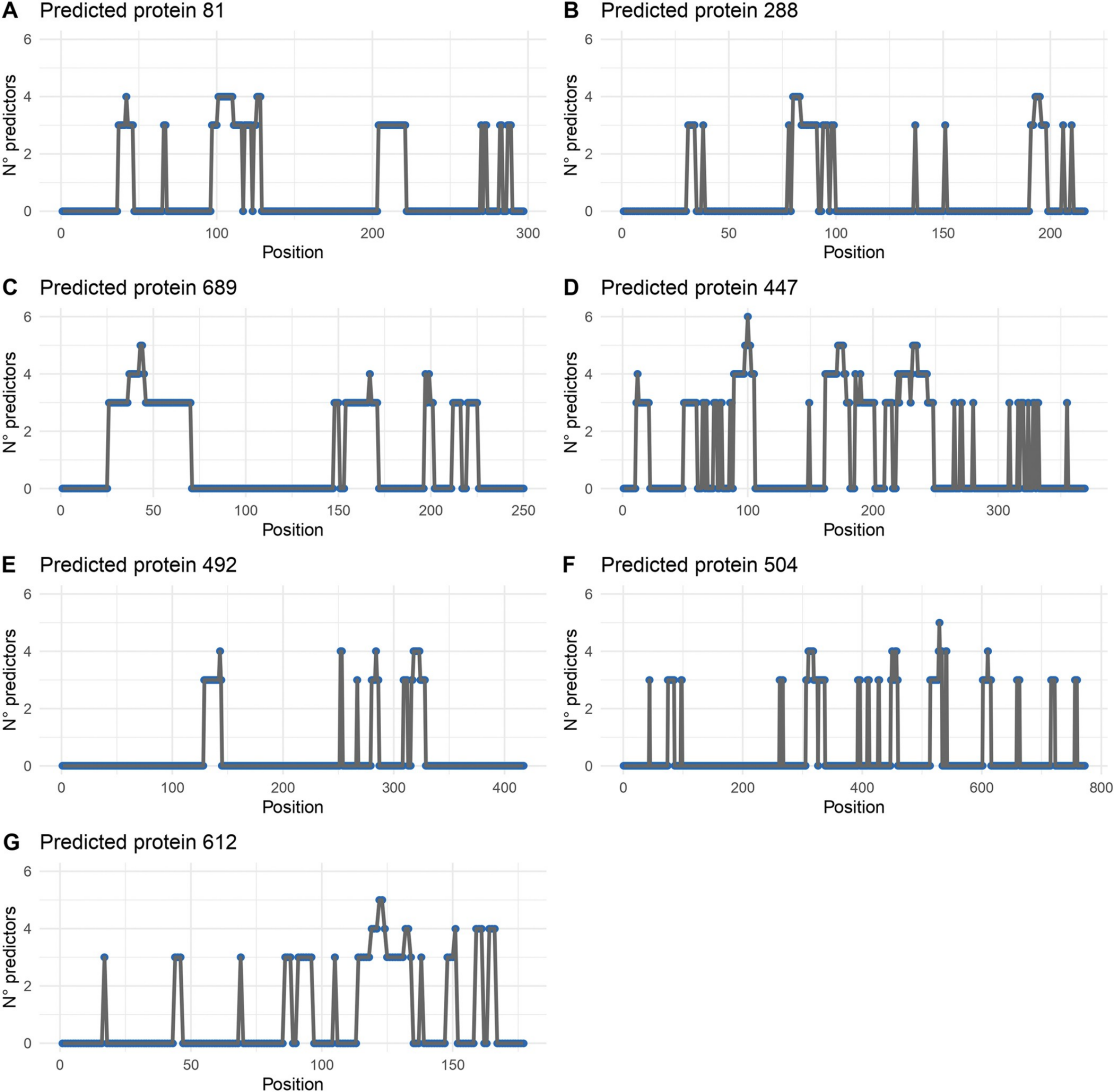
Schematic representation of linear B-cell predictions of the top seven proteins with more linear B-cell epitopes, generated with R library ggplot2.

Six web servers were employed to predict the linear epitopes in each *B*. *bacilliformis-*specific protein, the analysis was performed using signal-peptide-free sequences. Then, the number of predictions per position was plotted to facilitate the identification of protein regions with a higher number of epitopes. A general overview shows epitopes were predicted in more than 50% of the entire protein, except prot 492, see Fig 2. The top seven proteins displaying more linear B-cell epitopes were selected for further analysis.

**Fig 3:**
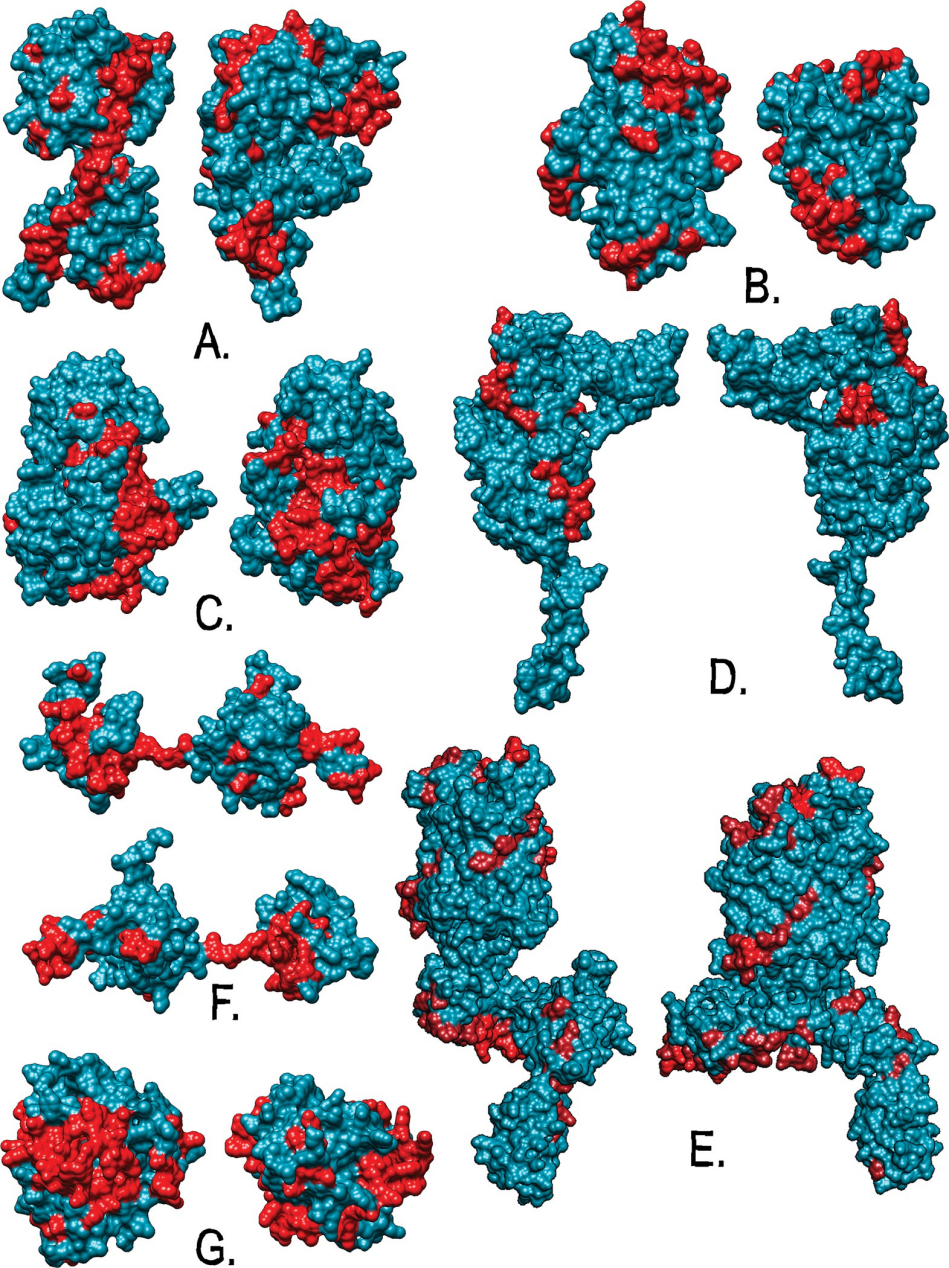
3D representation of the seven most promising proteins and their B-cell linear epitopes: A) Prot 81; B) Prot 288; C) Prot 447; D) Prot 492; E) Prot 504; F) Prot 612; G) Prot 689. The prediction was done by Phyre 2 and Protein models are displayed by using the software chimera. Colored regions represent epitopes predicted by at least three bioinformatics tool servers.

The linear B-cell epitopes were highlighted in the 3D simulated structure of the top seven proteins, Fig 3, no computational simulation calculations for lineal epitopes were obtained in 3D modeling. The 3D structure provides insight into the possible disposition of epitopes in the predicted proteins under the native state.

The identification of linear B-cell epitopes was performed by using six bioinformatics tools, the results were plotted for easy identification of areas that tend to contain more linear B-cell epitopes; this study considers as regions of interest those that were identified by at least 3 predictors, see Table 2 and Fig 2. Thus, we predicted that prot 447 is a peptidoglycan DD-metalloendopeptidase has regions highly enriched in linear B-cell epitopes, also, the prot 447 was the only protein that contain linear B-cell epitopes that were predicted by six tools. Likewise, regions highly enriched in linear B-cell epitopes were identified in prot 81, prot 288, prot 504, prot 492, prot 612, and prot 689. Prot 81 is predicted as a protein with tyrosine recombinase activity that participates in the integration of the genetic material of phages, prot 288 is predicted as a transport protein with ABC domain, prot 492 is predicted to be involved in cell division, prot 504 is predicted as a beta-barrel protein responsible for locating proteins in the outer membrane, prot 612 is predicted as a protein that participates in DNA replication and has a single-stranded DNA-binding domain, and prot 689 is predicted as a lipoprotein A with lytic transglycosylase activity. The size and number of the linear B-cell epitopes were variable in each protein, Table 2 shows the characteristic of epitopes.

**Table 2:** Proteins candidates of *B*. *bacilliformis*.

N°	Name	Protein lenght (size: amino acids)	Epitope location (amino acid positions)*	Epitope length (number of amino acids)*
1	Prot 81	322 aa	37–46; 97–116; 118–122; 124–128; 204–221	10aa; 20aa; 5aa; 5aa; 18aa
2	Prot 288	241 aa	31–34; 80–91; 191–198	4aa; 12aa; 8aa
3	Prot 447	397 aa	11–21; 49–59; 90–105; 162–181; 186–201; 210–215; 219–248	11aa; 11aa; 16aa; 20aa; 16aa; 6aa; 30aa
4	Prot 492	448 aa	129–144; 280–286; 309–312; 316–328	16aa; 7aa; 4aa; 13aa
5	Prot 504	798 aa	75–85;306–325; 327–337; 393–396; 448–459; 514–541; 602–615; 659–663; 716–722; 756–759	11aa; 20aa; 11aa; 4aa; 12aa; 28aa; 14aa; 5aa; 7aa; 4aa
6	Prot 612	177 aa	91–96; 114–138; 148–151	6aa,25aa; 4aa
7	Prot 689	281 aa	26–70; 154–171; 197–201; 212–216; 220–225	45aa; 18aa; 5aa; 5aa; 6 aa

* Minimum size of region of interest: 4aa

## Discussion

The *in-silico* identification of linear B-cell epitopes in specific proteins of *B*. *bacilliformi* proteins for their application in immunological methods is a strategy considered by several researchers, to solve the need for serological diagnosis. For the development of this study, the bioinformatic analysis aimed to identify non-homologous proteins with *Homo sapiens*, *Mus musculus*, other *Bartonellae*, and a subset of bacterial agents of febrile syndromes. This approach shares similarities with the methodology used by Ditcher *et al*., who identified potentially immunoreactive proteins by predicting putative antigenic proteins *in-silico* from the genomic sequences of *B bacilliformis*, evaluating homologies affecting potential cross-reactivities with other *Bartonella* spp [33]. All proteins identified in this study present percentages of identity less than 80% at the sequence level with respect to other species of the *Bartonella* genus and less than 70% with respect to other genera associated with fever. The *in-silico* identification of linear B-cell epitopes in specific proteins of *B*. *bacilliformis* was carried out using six epitope predictor tools available on web servers, the same ones that reside in algorithms such as supporting vector machine, SVM; random forest, RF; neural network, NN and physicochemical characteristics.

The performance of these methodologies has been analyzed simultaneously in different studies [34,35], and applied to different gram-negative bacteria such as *Coxiella burnetii* [36], *Vibrio vulnificus* [37], *Treponema pallidum* [38], *Anaplasma phagocytophilum* [39], among others. Furthermore, many of the proteins or peptides obtained in those studies have shown their utility in serological assays and/or in the generation of antibodies in murine models for the development of vaccines. In that regard, Dichter *et al*. identified three immunodominant proteins which were evaluated by ELISA and displayed a sensitivity of 81% and specificity of 95% when a porin B and an autotransporter E are combined [33]. Likewise, Padilla *et al*. show a protein as a vaccine candidate, designed using predicted epitopes by *in-silico* analysis [40]. The *in-silico* analysis allowed the identification of seven specific proteins of *B*. *bacilliformis*. The prot 81 was predicted as a phage integrase whose cellular localization is cytoplasmic. However, it did not discount the possibility to investigate this protein, since the ELISA assay performed at National Institute of Health-Peru is produced using total proteins of *B*. *bacilliformis* strains. The 228 protein is predicted to belong to the ABC (ATP-binding protein) transporter family containing three continuous linear epitopes based on analysis (Table 2). The predicted region of this protein is involved in metal cation exchange, according GenBank web, but so far it has not been characterized for *Bartonella bacilliformis* species. However, Hina et al identified a protein from the membrane transporter family (Hemin ABC transporter) as a candidate for the design of vaccines against *Bartonella bacilliformis*, using bioinformatic analysis [41].

A similar approach using *in-silico* analysis was performed by Dichter *et al*. who employed only the Vaxign software, they identified the peptidoglycan-binding protein (LysM) [33], the same one that we described in our study as 447. In our research, prot 447 was predicted to display 7 linear epitopes (6–30 amino acids), see Table 2, and minor sections that could not be considered as epitopes due to their size (2Aa to 5Aa). According to previous studies, the aggrupation of linear epitopes and minor sections could be part of a conformational epitope [42,43], likewise, it has been suggested that conformational epitopes are to be formed by linear epitopes [44,45]. It should be noted that unlike Dichter, in our study, more than three tools coincided in the same region in the prediction of epitopes within this protein, which supports our results and that 447 is considered a good antigenic candidate for the development of serological diagnostic methods.

Furthermore, a previous study has identified a homologous protein of Prot 447, the identification was done by screening heterologous proteins with serums of patients [46]. Also, the homologous protein of prot 447 has been identified in western-blot assays [46] and has been expressed *in-vitro* in *Escherichia coli* for ELISA-type serological assays [40]. In relation, Prot 447 identified in this study has been predicted as a 43kDa lipoprotein with metallopeptidase activity and a lysine domain responsible for peptidoglycan binding, as well as the previous study. The findings about the homologous of Prot 447 support our bioinformatics analysis, considering that the prot 447 predicted for us displayed several linear B-cell epitopes.

Likewise, prot 504 was predicted to be an outer membrane protein with a beta-barrel domain, this kind of protein has been shown to generate immunity against *Pasteurella multocida* [47], and to have a potential use as vaccine against *Haemophilus influenzae* [48,49] and *Leptospira* sp. [50]. The remaining five identified proteins are predicted to be involved in cell division, peptidoglycan binding, or transglycosylases, additionally, those proteins have not been reported previously and experimental analysis is required.

The finding of specific outer membrane proteins (OMPs) of *B*. *bacilliformis*, such as the lipoprotein prot 447 and the prot 504 with beta-barrels domain, provides the basis for the future implementation of an accurate and sensitive serological assay for the diagnosis of CD as OMPs can activate the immune response and virulence by mediating pathogen-host interactions [51]. In Gram-negative bacteria, the tertiary structure of OMPs includes beta-barrel structure composed of a variable number of beta-strands. Another type of exposed OMPs known as outer membrane lipoproteins are also involved [52,53]. In addition, both outer-membrane beta-barrels (OMBB) and outer-membrane lipoproteins (OMLP) are considered good candidates for the development of vaccines, immunological target tests and could promote better understanding of the pathogenicity of *B*. *bacilliformis*, facilitating the identification of therapeutic molecules for *in-silico* and experimental assays [54].

The prot 612 predicted as a single-strand binding protein has not previously been described, the available information on GenBank web points out this protein could be involved in DNA replication, DNA reparation, and DNA recombination. Also, the prot 689 was predicted to be a lytic transglycosylase, as is mentioned by GenBank web, septal ring lytic transglycosylase RlpA family (rare lipoprotein A). This kind of protein was studied in Gram-negative bacteria and was described to participate in division of cells [55], hence it can be inferred that prot 689 has a major role in bacterial survival. No serological analysis was performed using homologous proteins of prot 612 and prot 689, in previous studies.

### Perspectives and implications of this study

Adequate medical interventions rely on disease diagnosis. CD includes an acute phase, characterized by anemia and febrile illness and a chronic phase distinguished by skin eruptions [2]. Furthermore, the presence of asymptomatic carriers has been reported, being considered as reservoirs and sources of the disease [2,10]. It is therefore important for a serological test for CD to not only be sensitive and specific, but it should also be able to detect early (acute) and late (chronic) infections, as well as asymptomatic carriers. Detection of the latter is of special interest to advance towards the eradication of CD [2]. The development and implementation of IgM (acute phase) and IgG (chronic phase and previously exposed population) antibody tests, respectively is important, with ELISA, Immunoblot and immunochromatographic lateral flow being good alternatives. The validation of these methods should include the geographic representation of CD from different endemic areas of Ecuador and Peru.

Many of the *B*. *bacilliformis* proteins identified in this study could be used as targets since their high specificity (inhibiting the secretion of virulence proteins, inhibiting the maintenance of cell wall) or vaccines candidates (by designing a mix of complete antigens, multiepitope proteins, or outer-membrane vesicles containing virulence factors). Future studies should consider both 1) the genetic variability of *B*. *bacilliformis*, and 2) the genetic variability of the human immune components (HLA-I, HLA-II) and should be carried out using molecular dynamics approaches.

## Conclusions

We report the *in-silico* identification of proteins with a high number of predicted linear B-cell epitopes of *B*. *bacilliformis* by exploring a combination of predictors at the genome level. The list of seven protein candidates identified in this study could be used for the development of serological diagnostic tests, the production of monoclonal antibodies and the development of vaccine candidates for the control of CD.
